# Organic photovoltaic cell with 17% efficiency and superior processability

**DOI:** 10.1093/nsr/nwz200

**Published:** 2019-12-05

**Authors:** Yong Cui, Huifeng Yao, Ling Hong, Tao Zhang, Yabing Tang, Baojun Lin, Kaihu Xian, Bowei Gao, Cunbin An, Pengqing Bi, Wei Ma, Jianhui Hou

**Affiliations:** State Key Laboratory of Polymer Physics and Chemistry, Beijing National Laboratory for Molecular Sciences, CAS Research/Education Center for Excellence in Molecular Sciences, Institute of Chemistry, Chinese Academy of Sciences, Beijing 100190, China; State Key Laboratory of Polymer Physics and Chemistry, Beijing National Laboratory for Molecular Sciences, CAS Research/Education Center for Excellence in Molecular Sciences, Institute of Chemistry, Chinese Academy of Sciences, Beijing 100190, China; State Key Laboratory of Polymer Physics and Chemistry, Beijing National Laboratory for Molecular Sciences, CAS Research/Education Center for Excellence in Molecular Sciences, Institute of Chemistry, Chinese Academy of Sciences, Beijing 100190, China; University of Chinese Academy of Sciences, Beijing 100049, China; State Key Laboratory of Polymer Physics and Chemistry, Beijing National Laboratory for Molecular Sciences, CAS Research/Education Center for Excellence in Molecular Sciences, Institute of Chemistry, Chinese Academy of Sciences, Beijing 100190, China; State Key Laboratory for Mechanical Behavior of Materials, Xi’an Jiaotong University, Xi’an 710049, China; State Key Laboratory for Mechanical Behavior of Materials, Xi’an Jiaotong University, Xi’an 710049, China; State Key Laboratory of Polymer Physics and Chemistry, Beijing National Laboratory for Molecular Sciences, CAS Research/Education Center for Excellence in Molecular Sciences, Institute of Chemistry, Chinese Academy of Sciences, Beijing 100190, China; University of Chinese Academy of Sciences, Beijing 100049, China; State Key Laboratory of Polymer Physics and Chemistry, Beijing National Laboratory for Molecular Sciences, CAS Research/Education Center for Excellence in Molecular Sciences, Institute of Chemistry, Chinese Academy of Sciences, Beijing 100190, China; University of Chinese Academy of Sciences, Beijing 100049, China; State Key Laboratory of Polymer Physics and Chemistry, Beijing National Laboratory for Molecular Sciences, CAS Research/Education Center for Excellence in Molecular Sciences, Institute of Chemistry, Chinese Academy of Sciences, Beijing 100190, China; State Key Laboratory of Polymer Physics and Chemistry, Beijing National Laboratory for Molecular Sciences, CAS Research/Education Center for Excellence in Molecular Sciences, Institute of Chemistry, Chinese Academy of Sciences, Beijing 100190, China; State Key Laboratory for Mechanical Behavior of Materials, Xi’an Jiaotong University, Xi’an 710049, China; State Key Laboratory of Polymer Physics and Chemistry, Beijing National Laboratory for Molecular Sciences, CAS Research/Education Center for Excellence in Molecular Sciences, Institute of Chemistry, Chinese Academy of Sciences, Beijing 100190, China; University of Chinese Academy of Sciences, Beijing 100049, China

**Keywords:** organic photovoltaic cells, power conversion efficiency, scalable large-area production, processability, non-fullerene acceptor

## Abstract

The development of organic photoactive materials, especially the newly emerging non-fullerene electron acceptors (NFAs), has enabled rapid progress in organic photovoltaic (OPV) cells in recent years. Although the power conversion efficiencies (PCEs) of the top-performance OPV cells have surpassed 16%, the devices are usually fabricated via a spin-coating method and are not suitable for large-area production. Here, we demonstrate that the fine-modification of the flexible side chains of NFAs can yield 17% PCE for OPV cells. More crucially, as the optimal NFA has a suitable solubility and thus a desirable morphology, the high efficiencies of spin-coated devices can be maintained when using scalable blade-coating processing technology. Our results suggest that optimization of the chemical structures of the OPV materials can improve device performance. This has great significance in larger-area production technologies that provide important scientific insights for the commercialization of OPV cells.

## INTRODUCTION

Organic photovoltaic (OPV) technology is a promising candidate in use of sustainable solar energy; the power conversion efficiency (PCE) is growing very fast with great potential in practical applications [1–5]. In the last 30 years, development of new materials, optimization of device processing methods and blend morphology [6–12], and an improved understanding of device physics have greatly contributed to progress in OPV cells [13–15]. One of the biggest advantages of OPV cells is solution processability, facilitating large-area production at low-cost via scalable printing technologies [16–19]. Although the PCEs of single-junction OPV cells have surpassed 16% [20–22], most of the devices with cutting-edge performance were fabricated by spin-coating methods at small areas below 0.1 cm^2^, which is far away from practical applications. Furthermore, the spin-coating method is highly wasteful of solution, and is not suitable for large-scale production. Therefore, when designing highly efficient OPV materials, their applicability in scalable fabrication technologies over relatively large active areas must be investigated.

Recent achievements in OPV cells are dominated by development and application of non-fullerene acceptors (NFAs) [23–25]. High-performance NFAs show broad absorption from 400 to 900 nm [5], leading to efficient harvesting of solar photons and thus a high output current density. NFA-based devices show both reduced radiative and non-radiative energy losses (*E*_loss_s), having the benefit of obtaining high voltages [26–28]. PCEs of over 16% were obtained with NFA-based OPV cells. We note that most NFAs consist of fused five- or six-membered heterocycles. For instance, highly efficient NFAs such as ITIC [29], Y6 [5] and their derivatives have highly fused ladder-type structures. The large conjugated structure is beneficial to form ordered intermolecular π–π stacking and improve the charge transport [30–32]. However, the same feature results in poor solubility of the NFAs, making solution-processing procedures difficult. To solve this issue, fine-tuning the flexible side chains of NFAs is crucial in balancing the charge transport and solution processability. This is particularly important when scaling up the active area of the OPV cells because the device performance relies strongly on a uniform morphology [33–35]. The best large-area OPV cells using printing methods have a PCE of only 13% [36], which is far behind that of small-area spin-coated devices.

Here, we conduct side-chain engineering on a highly efficient NFA BTP-4Cl and study the applications of the OPV materials under different processing conditions. This approach shows improved photovoltaic performance for OPV cells with large-area fabrication. Impressively, the best device yields a maximum PCE of 17.0% at an active area of 0.09 cm^2^. This is among the top efficiencies for OPV cells, and the result has been certified by an independent institution. Importantly, when a blade-coating method was used to extend the active area of the active layer, a high PCE of 15.5% was maintained because of the balanced solution processability and charge transport. In comparison, the high efficiencies of the spin-coated OPV cells based on two other NFAs with shorter or longer alkyl chains suffered significant decreases when fabricating large-area devices using the blade-coating method.

## RESULTS AND DISCUSSION

**Figure 1.**
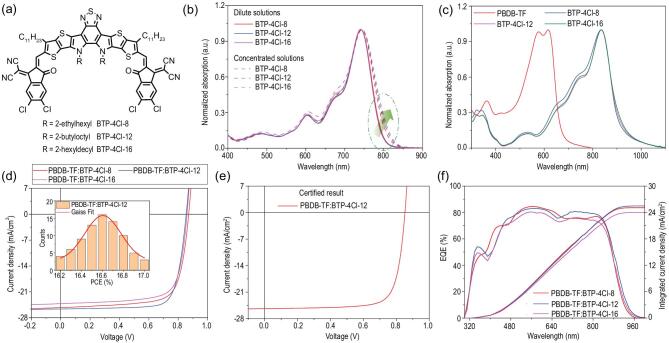
(a) Chemical structures of BTP-4Cl-X, where X represents 8, 12 or 16. (b) Normalized absorption spectra of BTP-4Cl-X in diluted (solid line) and concentrated (dashed line) chlorobenzene solutions. (c) Normalized absorption spectra of the neat donor and acceptors in thin films. (d) *J–V* curves of the best devices. The inset is a statistical diagram of PCEs for PBDB-TF:BTP-4Cl-12-based cells. (e) *J–V* curve of the OPV cell certified in the NIM. (f) EQE curves of the corresponding OPV cells.

In our recent work, we designed the chlorinated NFA BTP-4Cl and achieved superior photovoltaic efficiencies over Y6 in OPV cells, where PCEs of 16.1 ± 0.2% and 10.7 ± 0.5% were recorded using a spin-coating method at device areas of 0.09 and 1 cm^2^, respectively [21]. The high efficiencies of this material make it a good model to investigate the adaptability of scalable production technology in OPV cells. However, when we adopted the doctor blade-coating method to fabricate 0.81 cm^2^ devices, the PCE dropped dramatically to 10.7 ± 0.5% (Table 1 and Supplementary Fig. 1). This was mainly ascribed to the poor blend morphology caused by limited solubility of BTP-4Cl, as discussed below. As displayed in Fig. 1a, to improve the processability of BTP-4Cl (here named as BTP-4Cl-8 for comparison), we replaced the 2-ethylhexyl with longer side chains of 2-butyloctyl or 2-hexyldecyl and synthesized new NFAs BTP-4Cl-12 and BTP-4Cl-16, respectively. Detailed synthetic procedures and structural characterizations are provided in the Supplementary Data.

**Table 1.** Detailed photovoltaic parameters of the OPV cells.

Active layer	Coating method	*V*_OC_ (V)	*J*_SC_ (mA/cm^2^)	FF	PCE (%)^a^	Area (cm^2^)^b^

PBDB-TF:BTP-4Cl-8	Spin-coating	0.872	25.2	0.743	16.3 (16.1 ± 0.2)	0.06
	Spin-coating	0.863	24.9	0.711	15.3 (14.8 ± 0.3)	0.81
	Blade-coating	0.838	21.7	0.635	11.5 (10.7 ± 0.5)	0.81
PBDB-TF:BTP-4Cl-12	Spin-coating	0.858	25.6	0.776	17.0 (16.6 ± 0.2)	0.06
	Spin-coating^c^	0.853	25.4	0.772	16.7	0.06
	Spin-coating	0.849	25.5	0.738	16.0 (15.5 ± 0.3)	0.81
	Blade-coating	0.833	26.0	0.716	15.5 (14.9 ± 0.4)	0.81
PBDB-TF:BTP-4Cl-16	Spin-coating	0.862	24.2	0.748	15.6 (15.2 ± 0.2)	0.06
	Spin-coating	0.854	24.0	0.718	14.7 (14.2 ± 0.3)	0.81
	Blade-coating	0.807	19.4	0.689	10.8 (9.81 ± 0.6)	0.81

^a^The average parameters are calculated from more than 20 independent cells.

^b^The area of the mask; the device areas of small- and large-area OPV cells are 0.09 and 1.07 cm^2^, respectively.

^c^The result is obtained from NIM.

To investigate the molecular stacking properties, we measured the ultraviolet-visible (UV–vis) absorption spectra of the three NFAs in diluted and concentrated chlorobenzene solutions (Fig. 1b). In the dilute solution (∼ 5 μg/mL), the peak at 740 nm is highly determined by intramolecular charge transfer [37,38], and the change of alkyl chains has no significant effect. The absorption coefficients of the three NFAs were measured and the results are provided in Supplementary Fig. 2. With longer side chains, the NFAs show some increases in absorption coefficient, which may be related to enhanced intermolecular packing properties. When the concentration increases (∼10 mg/mL), the absorption is affected more by intermolecular charge transfer of the aggregators [37]. For the three NFAs, the absorption edges redshift with increasing alkyl chain length, which may imply enhanced aggregation properties in BTP-4Cl-12 and BTP-4Cl-16. Figure 1c shows the absorption spectra of the NFAs as thin films. We found that the main peaks of the three NFAs highly overlapped at 836 nm, a redshift of 90 nm over that in solution states. We measured the molecular energy levels of the three NFAs via electrochemical cyclic voltammetry measurements. As shown in Supplementary Fig. 3, the results suggest that modification of the side chains has little impact on the energy levels of the NFAs.

The crystalline properties of the NFAs were investigated by grazing-incidence wide-angle X-ray scattering (GIWAXS). Supplementary Fig. 4a shows the 2D GIWAXS patterns of the neat NFA films. The clear (010) diffraction peaks in the out-of-plane direction suggest that they have a preferential face-on orientation. Supplementary Fig. 4b presents the 1D profiles along the out-of-plane and in-plane directions. In the out-of-plane direction, the (010) diffraction peaks of BTP-4CL-8, BTP-4Cl-12 and BTP-4Cl-16 are located at 1.81, 1.84 and 1.74 Å^−1^, respectively, implying that BTP-4Cl-12 has the shortest π–π stacking distance. In the in-plane direction, we found that the lamellar packing distance increases with the longer alkyl chains. In addition, we also conducted the GIWAXS measurements on blend films based on PBDB-TF as donor (Supplementary Fig. 4c and d). The calculated (010) coherence length values are 2.18, 1.76 and 1.92 nm for BTP-4Cl-8-, BTP-4Cl-12- and BTP-4Cl-16-based devices, respectively. These results indicate that the PBDB-TF:BTP-4Cl-12-based blend film has the lowest crystalline property. The differences in crystalline properties may lead to varied microscopic morphologies.

To investigate the photovoltaic performance of BTP-4Cl-12 and BTP-4Cl-16, we first fabricated small area (0.09 cm^2^) spin-coated OPV cells, in which a conventional device structure of ITO/PEDOT:PSS/PBDB-TF [39]:NFA blend/PDINO/Al was adopted (ITO: indium tin oxide; PEDOT:PSS: poly(3,4-ethylenedioxythiophene): poly-(styrenesulfonate); PDINO [40]: perylene diimide functionalized with amino *N*-oxide). The device based on BTP-4Cl-8 was also prepared in parallel for clear comparison. The optimal device fabrication conditions based on the three NFAs are provided in the Supplementary Data.

Figure 1d shows the current density−voltage (*J−V*) curves of the optimized OPV cells, and the detailed photovoltaic parameters are collected in Table 1. In comparison, the variances in open-circuit voltages (*V*_OC_s) are very small. We carried out highly sensitive EQE and electroluminescence (EL) quantum efficiency (EQE_EL_) measurements, and found that the three OPV cells have similar band gaps and *E*_loss_s (Supplementary Fig. 5 and Supplementary Table 1). The PBDB-TF:BTP-4Cl-8-based OPV cell shows a maximum PCE of 16.3% with a *V*_OC_ of 0.872 V, a short-circuit current (*J*_SC_) of 25.2 mA/cm^2^ and a fill factor (FF) of 0.743, which are consistent with previous results [21]. The PCE of the OPV cell based on PBDB-TF:BTP-4Cl-16 is lower than that of the PBDB-TF:BTP-4Cl-8-based device because of the decreased *J*_SC_. The BTP-4Cl-12-containing device shows improved *J*_SC_ and FF values relative to the other two devices, leading to the highest PCE of 17.0%. To the best of our knowledge, this is the highest value for the published single-junction OPV cells so far. The inset in Fig. 1d shows a PCE histogram of 80 devices based on PBDB-TF:BTP-4Cl-12 from eight batches, with an average value of 16.6 ± 0.2%. We then sent the best cell to the National Institute of Metrology (NIM, China) for certification. As shown in Fig. 1e and Supplementary Fig. 6, the optimal PCE obtained from NIM is 16.7%. After 500 h in the nitrogen atmosphere, the encapsulated devices maintain ∼85–90% of the initial efficiencies (Supplementary Fig. 7). Figure 1f shows the EQE curves of the optimal devices. It can be seen that the BTP-4Cl-12-based device shows higher EQE values than the other devices in most regions of 450–850 nm. The integrated current densities are 25.1, 25.4 and 24.0 mA/cm^2^ for BTP-4Cl-8-, BTP-4Cl-12- and BTP-4Cl-16-based devices, respectively, which show good consistency with the *J–V* measurements.

**Figure 2.**
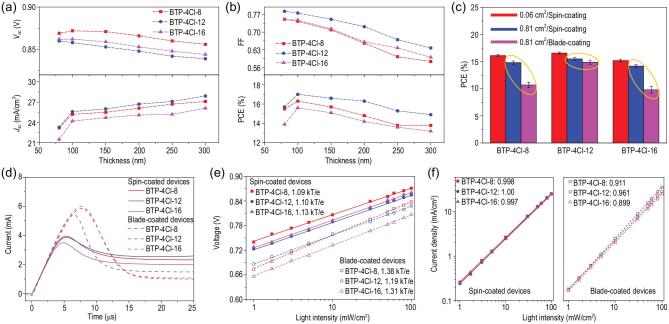
(a) *V*_OC_ and *J*_SC_ versus active layer thickness. (b) FF and PCE versus active layer thickness. (c) Statistics of OPV cell under different preparation conditions; the areas of the masks are shown in the panel. (d) Photo-CELIV curves of the devices. (e) *V*_OC_ of the devices as a function of light intensity. (f) *J*_SC_ of the devices against light intensity.

In addition to high efficiency, low sensitivity to thickness variation is important for practical production. As depicted in Fig. 2a and b, we studied the effect of active layer thickness on the photovoltaic characteristics (*V*_OC_, *J*_SC_, FF and PCE). The optimal thickness of the active layer is about 100 nm. As the active layer thickness is increased from 80 to 300 nm,

the *V*_OC_ and FF decrease. All the devices show some increase in *J*_SC_s for enhanced light absorption. As a result, all the devices can maintain >85% of optimal PCEs when the active layer thicknesses increase to 300 nm, which is beneficial to fabrication of large-area modules. In addition, there is no apparent difference in the three devices.

To explore the applicability of the OPV cells in large-area fabrication, we next adopted a blade-coating method to fabricate 1 cm^2^ devices. Fabrication procedures for the devices are described in the experimental part of the Supplementary Data. To better compare the spin-coating and blade-coating methods, we first fabricated the 1 cm^2^ devices using the spin-coating method. As shown in Fig. 2c and Supplementary Fig. 1, when extending the active area from 0.09 to 1 cm^2^, although all three devices show some decreases in photovoltaic parameters especially FF values, the PCEs are still above 14.5% (the detailed photovoltaic parameters are collected in Table 1). Impressively, a high PCE of 16.0% is recorded for the BTP-4Cl-12-containing OPV cell. For the blade-coated device based on PBDB-TF:BTP-4Cl-12, a maximum PCE of 15.5% was obtained, which is comparable to the spin-coated cell. It should be pointed out that the PCEs of both the spin-coated and blade-coated devices based on PBDB-TF:BTP-4Cl-12 are very pronounced results for OPV cells. In contrast, the BTP-4Cl-8- and BTP-4Cl-16-based cells suffer significant decreases in PCEs, with the best PCEs only around 11%. It is necessary to understand the reasons for the decline of photovoltaic performance for the blade-coating devices.

We studied the charge transport and recombination in the 1 cm^2^ devices fabricated by varied processing methods. First, we measured the mobilities of the fast carrier component by performing photo-CELIV measurements on the working devices (photo-CELIV: the photoinduced charge-carrier extraction in a linearly increasing voltage) [41]. As shown in Fig. 2d, when the spin-coating method was used, all the devices had similar mobilities: the calculated mobilities were 2.86 × 10^–5^, 2.92 × 10^–5^ and 3.10 × 10^–5^ cm^2^/V/s for BTP-4Cl-8-, BTP-4Cl-12- and BTP-4Cl-16-based devices, respectively. When the blade-coating technology was used, all the devices showed decreased mobilities to varying extents: the BTP-4Cl-12-based device showed a slight decrease (1.92 × 10^−5^ cm^2^/V/s), whereas remarkable decreases were observed in the devices based on BTP-4Cl-8 (9.23 × 10^−6^ cm^2^/V/s) and BTP-4Cl-16 (8.31 × 10^−6^ cm^2^/V/s). The lower mobilities will cause more charge recombination and thus decrease the *J*_SC_ and FF [42,43].

We then measured the *V*_OC_ and *J*_SC_ dependence on the incident light intensity (*P*_light_) for the different devices. The *V*_OC_ as a function of the light intensity is plotted in Fig. 2e. All the spin-coated devices show a weak dependence of *V*_OC_ on *P*_light_. The slope of Δ*V*_OC_*vs* Δln(*P*_light_) was used to investigate the trap-assisted recombination, where *k* is the Boltzmann constant, T is the absolute temperature and *q* is the electric charge [44–46]. The slopes were 1.09, 1.10 and 1.13 *k*T/*q* for the devices based on BTP-4Cl-8, BTP-4Cl-12 and BTP-4Cl-16, respectively. When the blade-coating method replaced the spin-coating method to fabricate the devices, all the devices showed increased slopes. The BTP-4Cl-12-based device showed a slightly higher slope of 1.19 *k*T/*q*, whereas much higher slopes of 1.38 and 1.31 *k*T/*q* were calculated for the BTP-4Cl-8- and BTP-4Cl-16-based devices. Under the same processing conditions, the lower slope of the BTP-4Cl-12-based device implies a more suppressed trap-assisted recombination in the devices. The significantly increased slopes are one of the main reasons for the decreased PCEs of the devices based on BTP-4Cl-8 and BTP-4Cl-16 [45].

The relationship between *J*_SC_ and *P*_light_ is plotted in Fig. 2f, where the exponential factor (s) of the power-law equation *J*_SC_ ∝ *P*_light_^s^ can reflect the degree of bimolecular recombination. For the 1 cm^2^ devices made by spin-coating method, we found that the *J*_SC_ exhibits almost linear dependence on the *P*_light_, implying a negligible bimolecular recombination in these devices [47]. When the blade-coating technology was used to fabricate the BTP-4Cl-12-based device, the s value decreased slightly to 0.961. In contrast, the BTP-4Cl-8- and BTP-4Cl-16-based devices yielded much lower *S* values of 0.911 and 0.899, respectively. These results suggest that bimolecular recombination is more pronounced in the blade-coated devices, which is associated with the lower charge mobilities.

**Figure 3.**
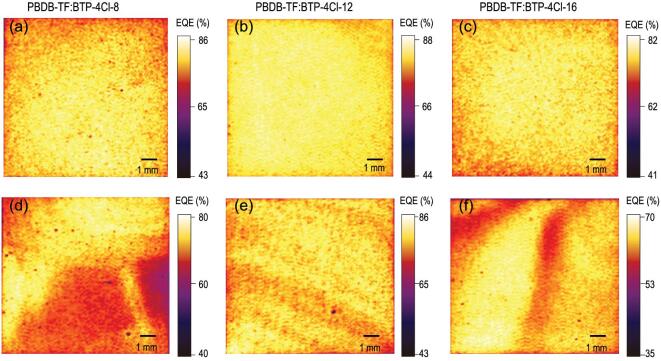
(a–c) The EQE mapping images of the OPV cells fabricated via spin-coating method. (d–f) The EQE mapping images of the OPV cells fabricated via blade-coating method.

From the above results, it can be reasonably concluded that higher charge transport and more suppressed charge recombination in the BTP-4Cl-12-based devices are the main reasons for the enhanced *J*_SC_s and FFs over the BTP-4Cl-8- and BTP-4Cl-16-based devices. To better understand how the processing technology affects the device performance, we first scanned the entire working area (1 cm^2^) via a 520 nm laser and mapped the EQE values, which can give a clear view of how the morphology affects the photon-response of the OPV cells. As presented in Fig. 3a–c, the EQE maps for the spin-coated devices are very uniform, which suggests that the whole regions have highly efficient charge generation, transport and collection. The high EQE values are consistent with their high *J*_SC_s in the *J–V* measurements.

Unlike the spin-coating method, drying wet film is difficult by using the blade-coating method. The solubility and aggregation properties of the active materials have a great impact on the blend morphology of the resulting films. For the blade-coated 1 cm^2^ OPV cells, the uniformity of the EQE maps is not as good as that of the spin-coated devices (Fig. 3d–f). For the BTP-4Cl-8-based blend film (Fig. 3d), the relatively low solubility of BTP-4Cl-8 makes it easily dissolve out from the solution, leading to a non-uniform film. For the blend film based on BTP-4Cl-16, good solubility and strong aggregation feature (Fig. 1b) may result in overlarge clusters. The BTP-4Cl-12-based device shows a relatively uniform EQE map without many low EQE regions.

**Figure 4.**
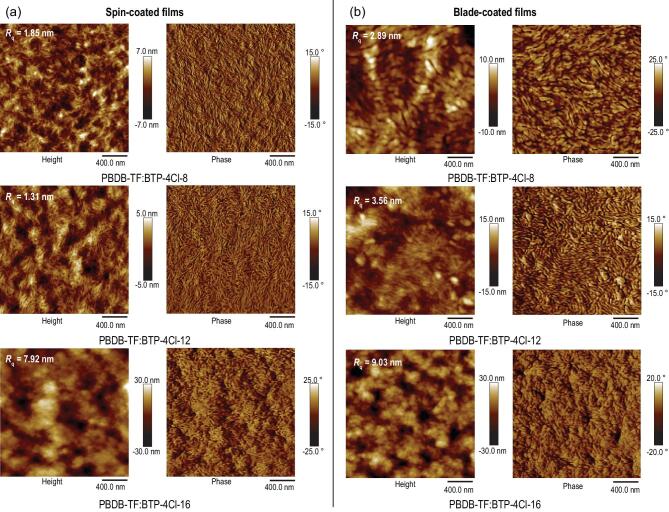
AFM height images and phase images of PBDB-TF:BTP-4Cl-X blend films prepared by (a) spin-coating process and (b) blade-coating method.

Furthermore, to get a more microscopic view, we studied differences in the surface morphology between the spin-coated and blade-coated photoactive layers using the atomic force microscopy (AFM). As shown in Fig. 4, the blend films based on PBDB-TF:BTP-4Cl-X fabricated by the different methods present remarkably different surface roughness and phase separation features. For the spin-coated films, the BTP-4Cl-8- and BTP-4Cl-12-based blend show a smooth surface and good phase separation features, and the mean-square surface roughness (*R*_q_) is 1.85 and 1.31 nm, respectively. In contrast, the *R*_q_ of the BTP-4Cl-16-based film is as large as 7.92 nm, which could be ascribed to its relatively low photovoltaic performance. The volatilization rate of the solvent decreased significantly when the

blade-coating method was used [48–50], leading to a longer time for ordered molecular alignment and aggregation. As illustrated in Fig. 4b, the *R*_q_ values and domain sizes increase for all the blade-coated films. For the blade-coated BTP-4Cl-12-based film, suitable phase separation with appropriate domain size is maintained, which may be attributed to the lower crystalline property. In comparison, larger domains are obtained for the BTP-4Cl-8- and BTP-4Cl-16-based blend films.

## CONCLUSION

In summary, aiming to improve the photovoltaic performance and processability of OPV cells, we performed side-chain engineering on the highly efficient NFA material and synthesized BTP-4Cl-X (X = 8, 12 or 16). By employing the polymer donor PBDB-TF, and the NFA BTP-4Cl-12, we successfully demonstrated a high PCE of 17% in single-junction OPV cells. As a result of the balanced solution processability and aggregation feature of BTP-4Cl-12, the blend film based on PBDB-TF:BTP-4Cl-12 showed very good morphology when the blade-coating method was used, contributing to high carrier transport, and suppressed charge recombination in the resulting OPV cell. Therefore, 1 cm^2^ OPV cells based on the blade-coating method yield a high PCE of 15.5%. These results are among the top values for OPV cells. This work provides important guidelines for developing highly efficient OPV materials by considering their applications in large-scale production.

## Supplementary Material

nwz200_Supplemental_File
