# Supplementary material for: Organic photovoltaic cell with 17% efficiency and superior processability
Source: Natl Sci Rev. 2019 Dec 5;7(7):1239–46. doi: 10.1093/nsr/nwz200 (PMC8288938; doi:10.1093/nsr/nwz200)
Supplement: nwz200_Supplemental_File [file nwz200_supplemental_file.docx]

## Supplementary material

17% efficiency organic photovoltaic cell with superior processability

Yong Cui^1^, Huifeng Yao^1,^*, Ling Hong^1,2^, Tao Zhang^1^, Yabing Tang^3^, Baojun Lin^3^, Kaihu Xian^1,2^, Bowei Gao^1,2^, Cunbin An^1^, Pengqing Bi^1^, Wei Ma^3^ and Jianhui Hou^1,2^

^1^State Key Laboratory of Polymer Physics and Chemistry, Beijing National Laboratory for Molecular Sciences, CAS Research/Education Center for Excellence in Molecular Sciences, Institute of Chemistry, Chinese Academy of Sciences, Beijing 100190, China

^2^University of Chinses Academy of Sciences, Beijing 100049, China

^3^State Key Laboratory for Mechanical Behavior of Materials, Xi’an Jiaotong University, Xi’an 710049, China

∗Corresponding authors. E-mails: yaohf@iccas.ac.cn

**Materials**

The core unit of BT-CHO was purchased from Solarmer Material Inc. The electron-accepting unit (Cl-EG) was synthesized according to our previous work [1]. Other reagents and solvents used were purchased from commercial sources and used as received.

**The synthesis of acceptors**.

**Supplementary Scheme 1.** The synthetic route of the BTP-4Cl-8, BTP-4Cl-12 and BTP-4Cl-16.

**BTP-4Cl-8:** BTP-4Cl-8 was synthesized according to our previous work.

**BTP-4Cl-12:** The synthetic route of BTP-4Cl-12 is similar to that of BTP-4Cl-8 [2], and the corresponding yield is about 54%. MS (MALDI-TOF) m/z: Calcd for C_90_H_102_Cl_4_N_8_O_2_S_5_: 1629.96; Found 1628.3. The MS, ^1^H NMR, and ^13^C NMR spectra are provided in Supplementary Figs. 8-10.

**BTP-4Cl-16:** The synthetic route of BTP-4Cl-16 is similar to that of BTP-4Cl-8 [2], and the corresponding yield is about 54%. MS (MALDI MALDI-TOF) m/z: Calcd for C_98_H_118_Cl_4_N_8_O_2_S_5_: 1742.18; Found 1741.6. The MS, ^1^H NMR, and ^13^C NMR spectra are provided in Supplementary Figs. 11-13.

**Device fabrication**

**Fabrication of the 0.09 cm^2^ and 1 cm^2^ device via spin-coating method**. Devices were fabricated with the conventional device structure of glass/ITO/PEDOT:PSS/ PBDB-TF:NFA blend/PDINO/Al. For the anode interlayers, PEDOT:PSS (4083) was purchased from the Clevios^TM^, and was diluted with the same volume of water. For the cathode interlayer, PDINO was purchased from Solarmer Material Inc, and was dissolved in methanol at the concentration of 1.5 mg/ml. For the active layer, PBDB-TF was purchased from Solarmer Material Inc. PBDB-TF:BTP-4Cl-8 (D/A 1:1.1), PBDB-TF:BTP-4Cl-12 (D/A 1:1.2) and PBDB-TF:BTP-4Cl-16 (D/A 1:1.2) were dissolved in chlorobenzene at the polymer concentration of 10 mg/ml. PBDB-TF:BTP-4Cl-8-based active layer solution should be stirred at 80˚C for at least 3 h. PBDB-TF:BTP-4Cl-12- and PBDB-TF:BTP-4Cl-16-based active layer solutions should be stirred at 40˚C. Before spin-coating the active layer, 2% 1-Chloronaphthalene (v/v) was added to the PBDB-TF:BTP-4Cl-8 solution, and 0.4% 1,8-diiodooctane (v/v) was added to the PBDB-TF:BTP-4Cl-12 and PBDB-TF:BTP-4Cl-16 solutions, respectively. Devices were fabricated by the following conditions: Firstly, about 10 nm PEDOT:PSS layers were spin-coated on the pre-cleaned ITO substrates and annealed at 150°C for 20 min. Subsequently, the substrates were transferred to the glove box. The mixed solutions were spin-coated onto the PEDOT:PSS layers, and then the films were treated with the thermal annealing at 100˚C for 10 min. The thickness of all active layers was about 100 nm. PDINO was spin-coated on the top of the active layers at 3000 rpm for 30 s. Finally, 200 nm Al was deposited under a high vacuum. The areas of the masks are about 0.0625 cm^2^ and 0.81 cm^2^ in our laboratory. The area of the mask is 0.06169 cm^2^ in NIM.

**Fabrication of the 1 cm^2^ device via blade-coating method**. The fabrication process of the blade-coated device is similar to that of the spin-coated device. The difference is the preparation of the active layer. For the blade-coated device, active materials were dissolved in CB at the polymer concentration of 4 mg/ml. Before spin-coating the active layer, 0.5% 1-Chloronaphthalene (v/v) was added to the PBDB-TF:BTP-4Cl-8 solution, and 0.1% 1,8-diiodooctane (v/v) was added to the PBDB-TF:BTP-4Cl-12 and PBDB-TF:BTP-4Cl-16 solutions, respectively. When coating the active layer, the blade moved at the speed of 5 cm/s. The ITO-based substrate is heated to 70°C. Subsequently, these films were treated with the thermal annealing at 100˚C for 10 min.

**Instruments and Measurements**

The *J*−*V* measurements were performed via the solar simulator (SS-F5-3A, Enlitech) along with AM 1.5G spectra whose intensity was calibrated by the certified standard silicon solar cell (SRC-2020, Enlitech) at 100 mW/cm^2^. The EQE spectrum was measured through the Solar Cell Spectral Response Measurement System QE-R3011 (Enli Technology Co., Ltd., Taiwan). Absorption spectra of all of the materials were measured on a Hitachi UH4150 UV-Vis spectrophotometer. Electrochemical cyclic voltammetry measurements were performed on a CHI650D electrochemical workstation with a three-electrode system. Pt wire and glassy carbon electrode were used as the counter electrode and working electrode, respectively. Ag/Ag^+^ was used as the reference electrode, and the ferrocene/ferrocenium redox couple (Fc/Fc^+^) was used as the internal calibration. Atomic force microscopy (AFM) height and phase images were obtained by a Nanoscope V AFM on tapping mode. In order to investigate the dependence of device performance on light intensity, neutral density filters were used to tune the light intensity, which was calibrated by the standard Si solar cell. The photo-CELIV measurements reported were performed by the all-in-one characterization platform Paios developed and commercialized by Fluxim AG, Switzerland. In order to reflect the true information, all of the devices are prepared for photoinduced charge extraction by linearly increasing voltage (photo-CELIV) measurements according to the corresponding device fabrication conditions. Ramp Rate: 0.10 V/us; Delay Time: 1.00 us; Light-Pulse Length: 100 us; Setup-Type: LED. The EQE mapping measurements were carried out by using LSD4 system (Enlitech). The instrument is equipped with 405 and 520 nm lasers. Considering the OPV cells have high EQE values around 520 nm, we select 520 nm laser under the test. Highly Sensitive EQE was measured by using an integrated system (PECT-600, Enlitech), where the photocurrent was amplified and modulated by a lock-in instrument. Electroluminescence (EL) quantum efficiency (EQE_EL_) measurements were performed by applying external voltage/current sources through the devices (ELCT-3010, Enlitech). GIWAXS measurements were conducted at 23A small- and wide-angle X-ray scattering (SWAXS) beamline at the National Synchrotron Radiation Research Center (NSRRC), Hsinchu, Taiwan. The wavelength of X-ray was 1.240 Å (10 keV) and the scattering signals were collected by a C9728DK area detector. The sample to detector distance was ≈101 mm, calibrated with a lanthanum hexaboride (LaB6) sample. The incident angle was kept at 0.14°.

**Supplementary Figure 1.** *J−V* curves of 1 cm^2^ OPV cells (the area of the mask is 0.81 cm^2^) based on PBDB-TF:BTP-4Cl-X fabricated via (a) spin-coating and (b) blade-coating methods.

**Supplementary Figure 2.** absorption coefficients of the three acceptors (a) in the CB solutions and (b) in the solid films.

**Supplementary Figure 3.** (a) Electrochemical cyclic voltammetry curves of the three acceptors; The cyclic voltammetry measurements don't show the difference of energy level between them. (b) Molecular energy levels of PBDB-TF and the three acceptors in this work.

**Supplementary Figure 4.** (a) 2D GIWAXS images of BTP-4Cl-X-based films. (b) The corresponding 1D profiles along the in-plane and out–of–plane directions. (c) 2D GIWAXS images of PBDB-TF:BTP-4Cl-X-based films fabricated via spin-coating method. (d) The corresponding 1D profiles along the in-plane and out–of–plane directions; The π-π stacking distances of PBDB-TF:BTP-4Cl-8, PBDB-TF:BTP-4Cl-12 and PBDB-TF:BTP-4Cl-16 are 3.57, 3.55 and 3.61 Å, respectively. Empirically, a negative correlation is observed between π-π stacking distance and device performance. In particular, PBDB-TF:BTP-4Cl-12-based device exhibits the shortest stacking distance and achieves the highest PCE.

**Supplementary Figure 5.** (a-c) Normalized EQE and EL spectra of PBDB-TF:BTP-4Cl-X-based devices; *E*_g_ depends on the intersection point. (d) The e*V*SQ OC and Δ*E*_1_ plotted as a function of the band gap. (e) Highly sensitive EQE curves of both devices. (f) The EQEEL of PBDB-TF:BTP-4Cl-X-based devices

**Supplementary Table 1.** Detailed energy losses of the three devices.

| Device | *E*_g_ (eV) | *qV*_OC_ (eV) | Δ*E*_1_ (eV) ^a)^ | Δ*E*_2_ (eV) ^b)^ | Δ*E*_3_ (eV) ^c)^ | *E*_loss_ (eV) |
| --- | --- | --- | --- | --- | --- | --- |
| BTP-4Cl-8 | 1.406 | 0.872 | 0.263 | 0.049 | 0.219 | 0.534 |
| BTP-4Cl-12 | 1.391 | 0.858 | 0.262 | 0.050 | 0.225 | 0.533 |
| BTP-4Cl-16 | 1.399 | 0.862 | 0.262 | 0.055 | 0.217 | 0.537 |

^a)^ $\Delta E_{1}= E_{g}- qV_{\mathrm{OC}}^{\mathrm{SQ}}$; Δ*E*_1_ is attributed to radiative recombination from the absorption above the bandgap. Where $V_{\mathrm{OC}}^{\mathrm{SQ}}$is the maximum voltage by the Shockley–Queisser limit; *q* is the elementary charge.

^b)^$\Delta E_{2}= qV_{\mathrm{OC}}^{\mathrm{SQ}}- qV_{\mathrm{OC}}^{\mathrm{rad}}$; Δ*E*_2_ is attributed to additional radiative recombination from the absorption below the bandgap. Where $V_{\mathrm{OC}}^{\mathrm{rad}}$is the open-circuit voltage when there is only radiative recombination.

^c)^$\Delta E_{3}= qV_{\mathrm{OC}}^{\mathrm{non}-\mathrm{rad}}= -kTln\left( \mathrm{EQE}_{\mathrm{EL}} \right)$; Δ*E*_3_ is non-radiative recombination.


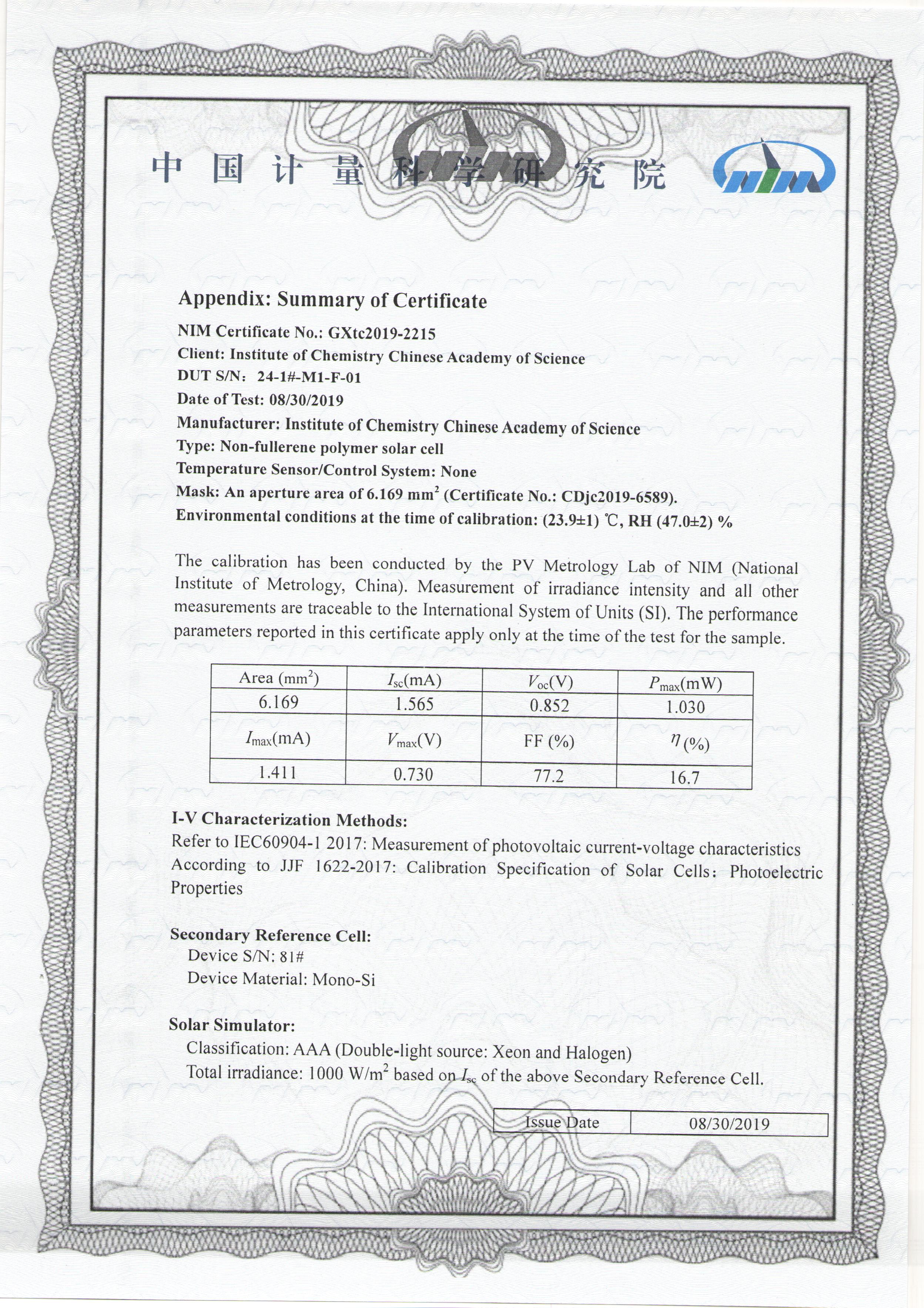


**Supplementary Figure 6.** Certification of the device performance from NIM, China.

**Supplementary Figure 7.** The long-term stability (h). These devices were encapsulated and intermittently tested by using a solar simulator (SS-F5-3A, Enlitech) along with AM 1.5G spectra. The test was carried out at a temperature of about 25°C in the glove box. There is no clear effect on long-term stability by changing side chain.


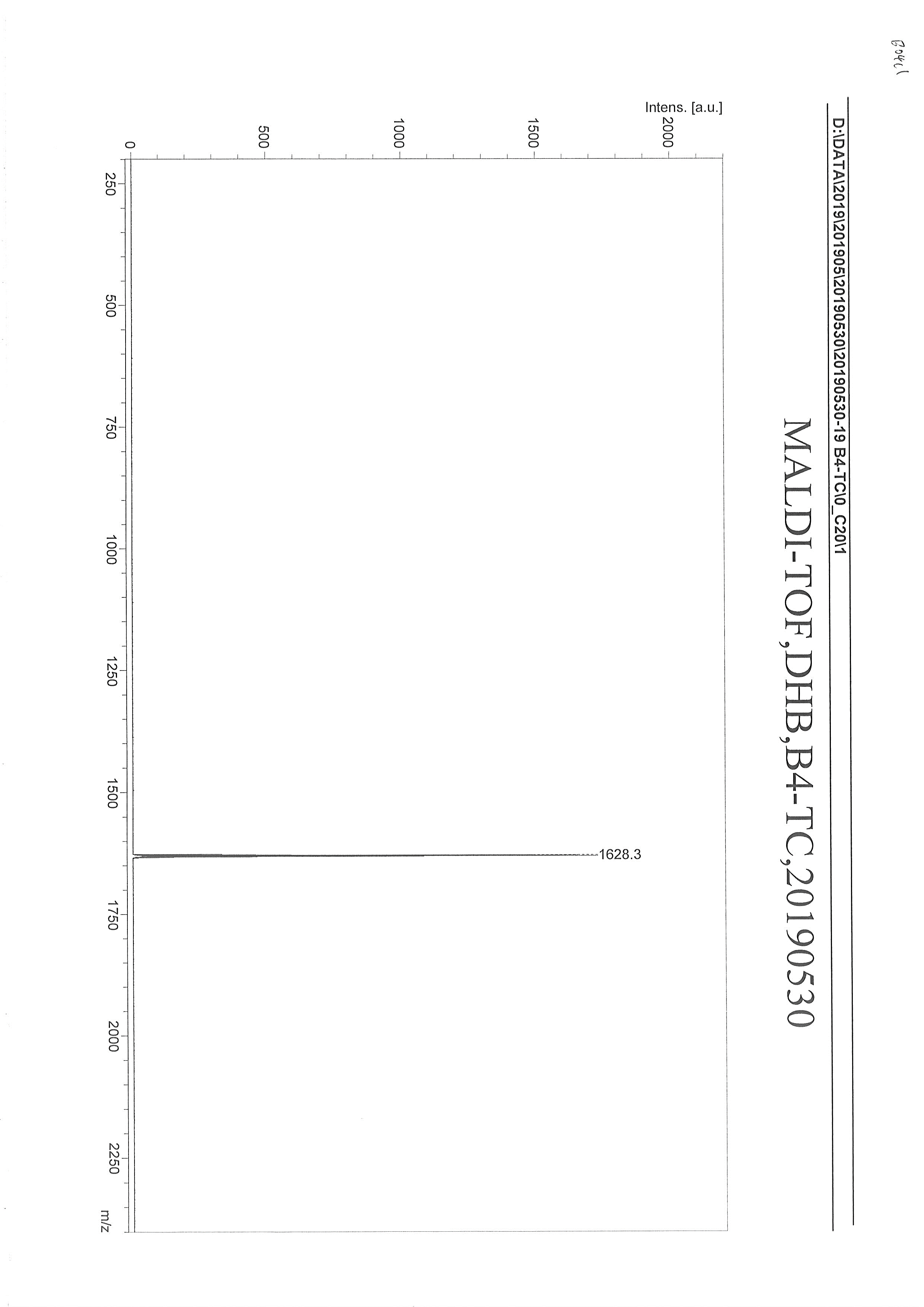


**Supplementary Figure 8.** MS spectrum of BTP-4Cl-12.


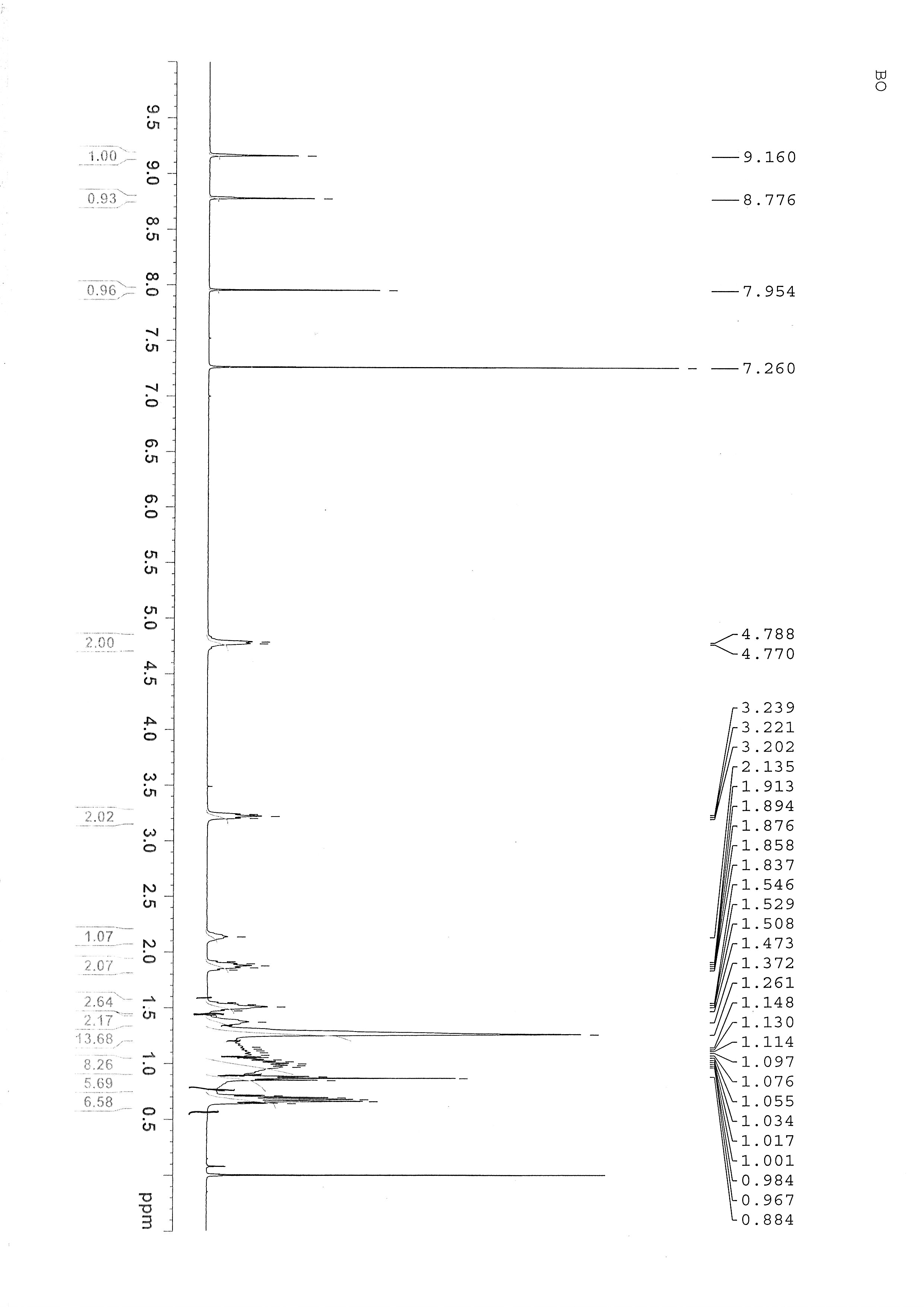


**Supplementary Figure 9.** ^1^H NMR spectrum of BTP-4Cl-12.


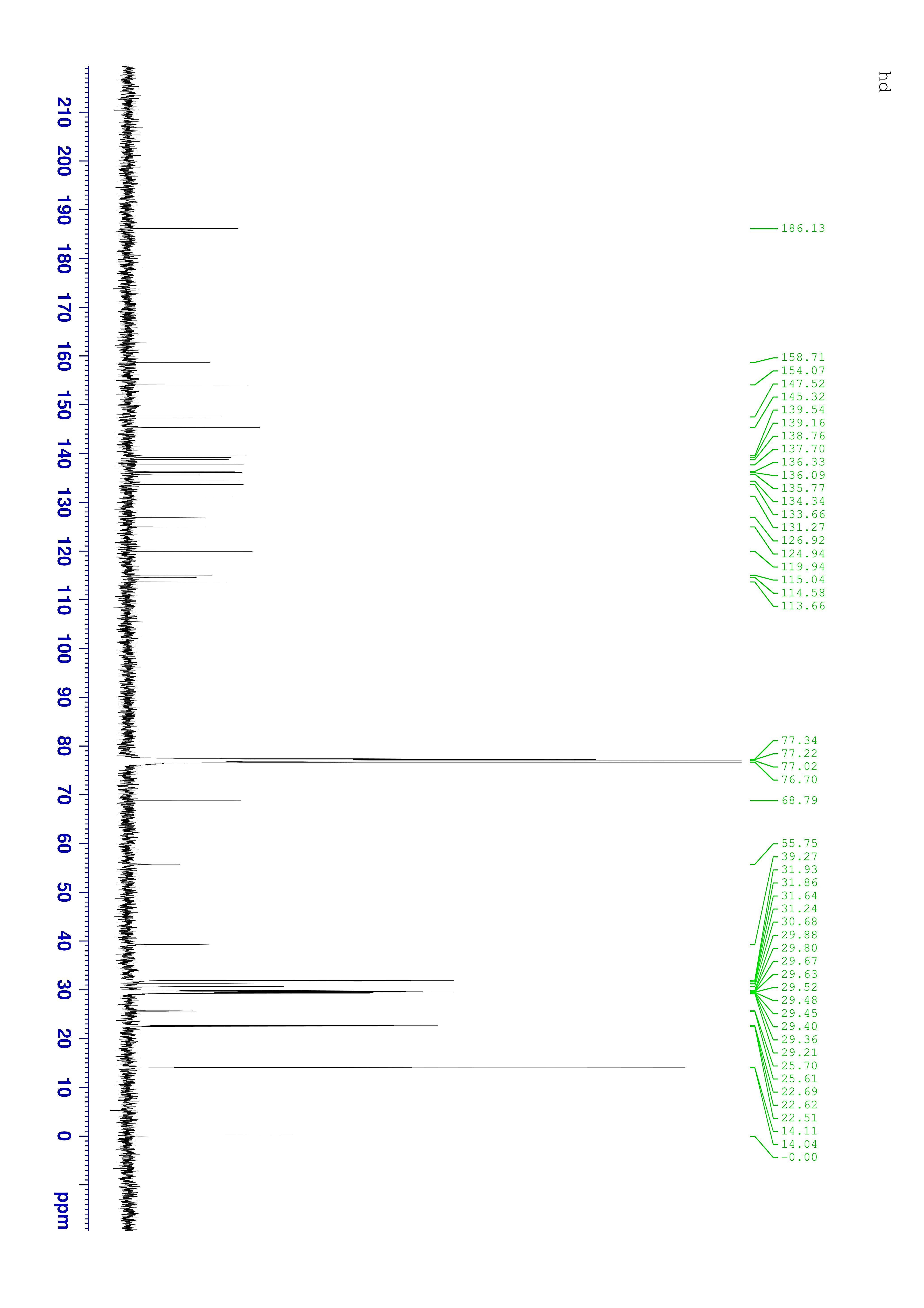


**Supplementary Figure 10.** ^13^C NMR spectrum of BTP-4Cl-12.


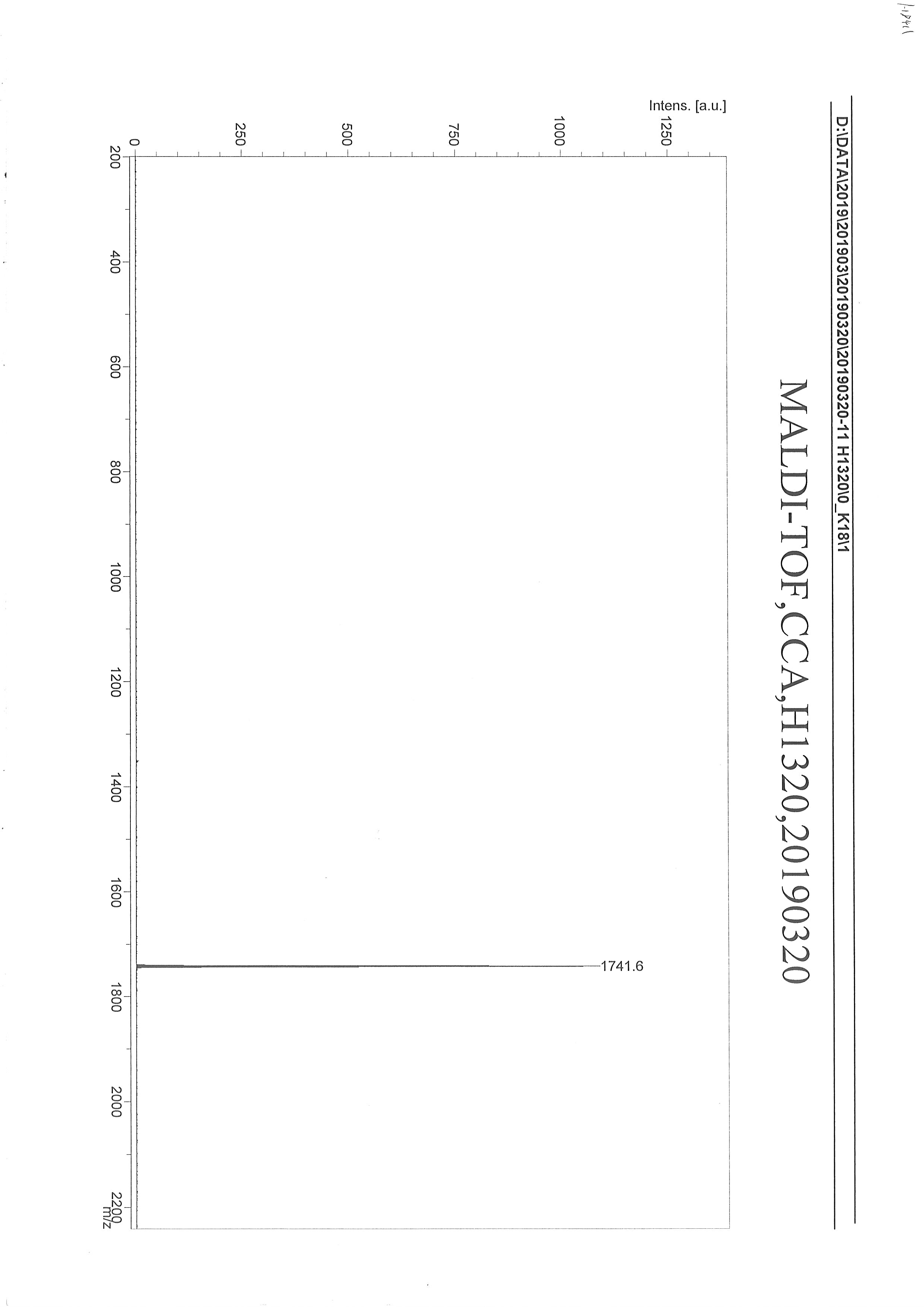


**Supplementary Figure 11.** MS spectrum of BTP-4Cl-16.


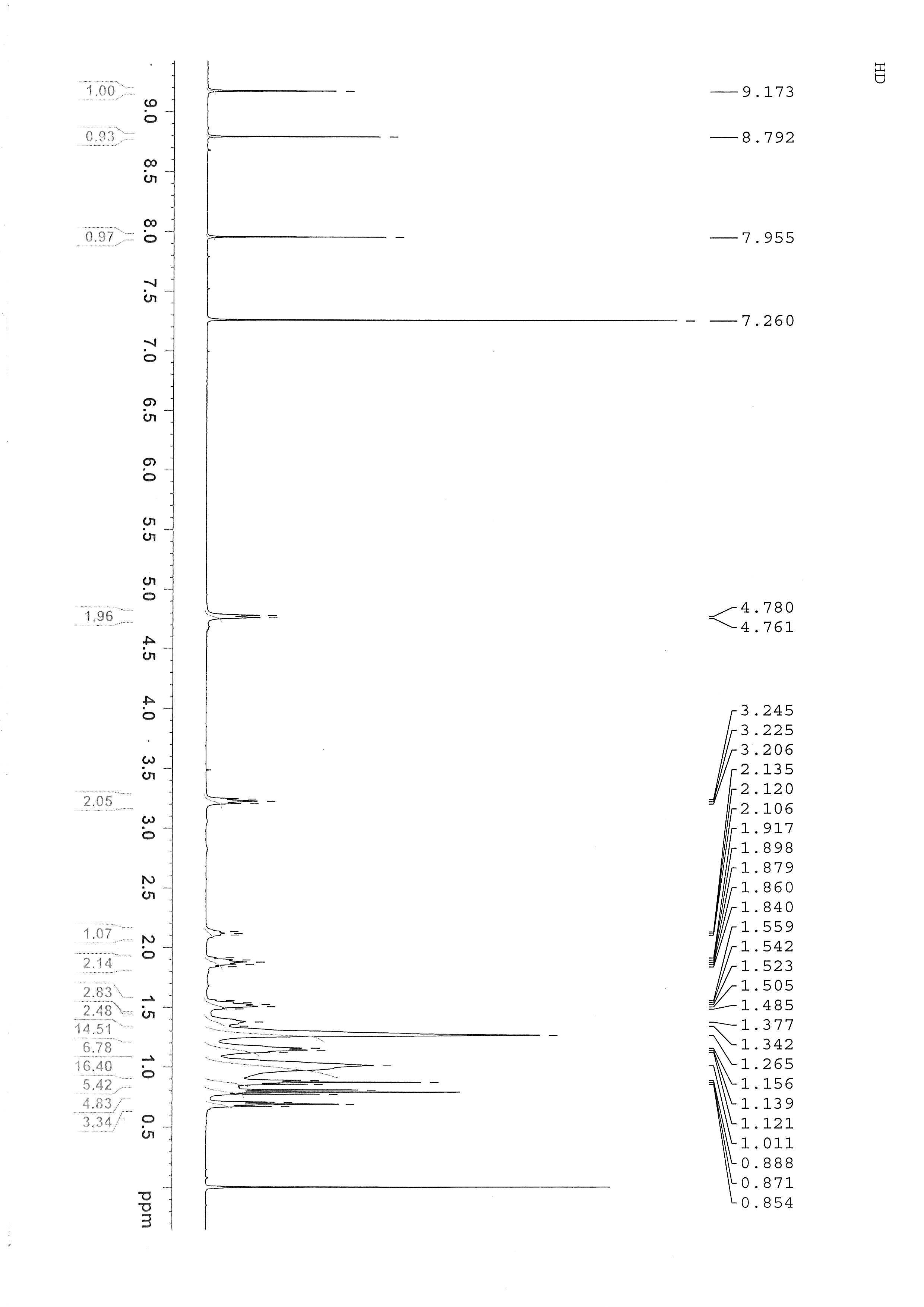


**Supplementary Figure 12.** ^1^H NMR spectrum of BTP-4Cl-16.


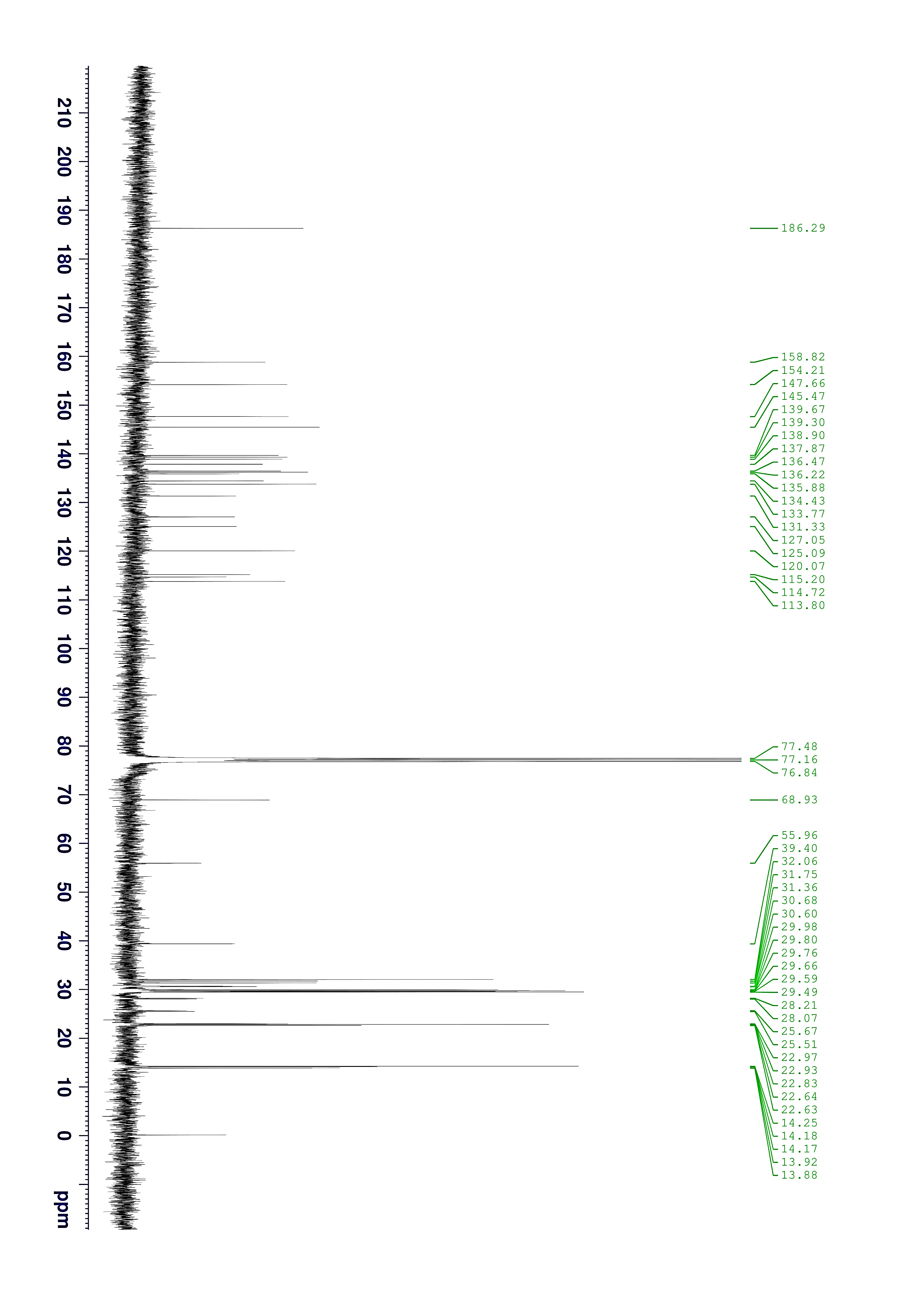


**Supplementary Figure 13.** ^13^C NMR spectrum of BTP-4Cl-16.

**References**

1. Cui Y, Yang C and Yao H *et al.* Efficient Semitransparent Organic Solar Cells with Tunable Color enabled by an Ultralow-Bandgap Nonfullerene Acceptor. *Adv Mater* 2017; **29**: 1703080.

2. Cui Y, Yao H and Zhang J *et al.* Over 16% efficiency organic photovoltaic cells enabled by a chlorinated acceptor with increased open-circuit voltages. *Nat Commun* 2019; **10**: 2515.
